# Biomarker identification of chronic atrophic gastritis and its potential drug analysis

**DOI:** 10.3389/fgstr.2022.948323

**Published:** 2022-09-23

**Authors:** Biao Song, Qinglin Cao, Tingting Li, Yun Liu, Qin Sun, Shanshan Fan, Xuejun Li

**Affiliations:** ^1^ The Graduated School, Anhui University of Traditional Chinese Medicine, Hefei, China; ^2^ Department of Gastroenterology, The Second Affiliated Hospital of Anhui University of Traditional Chinese Medicine, Hefei, China; ^3^ The Graduated School, Anhui Normal University, Wuhu, China

**Keywords:** chronic atrophic gastritis, GEO database, tissue specific, biomarker, diagnosis

## Abstract

**Background:**

Chronic atrophic gastritis (CAG) is the first step of gastric precancerous lesions, and the study of the pathogenesis of CAG is helpful for the prevention and treatment of gastric cancer(GC). The purpose of this study is to explore the potential biomarkers and therapeutic drugs of CAG through bioinformatics analysis.

**Methods:**

The GSE11632 dataset was downloaded from Gene Expression Omnibus (GEO) database and the differentially expressed genes (DEGs) were obtained by using GEO2R online tool. We searched GeneCard and DisGeNET databases for genes related to CAG and used the overlapping genes as final DEGs for further functional enrichment analysis and Protein-protein Interaction (PPI) network analysis. Tissue-specific expressed genes were identified by BioGPS database. Cytoscape software was used to identify key hub genes and validated them in GSE27411 data sets. The upstream miRNAs of hub gene was predicted by TargetScan, miRDB and miRWalk. Finally, run the Connectivity Map (CMap) to identify new potential drugs for the treatment of CAG.

**Results:**

A total of 430 differentially expressed mRNA were identified in this study, including 315 up-regulated genes and 115 down-regulated genes. After intersecting with CAG-related genes in GeneCard and DisGeNET databases, 42 DEGs were obtained. 24 DEGs were identified as tissue-specific expressed genes, most of which were expressed in stomach. GO and KEGG pathway analysis showed that DGEs was mainly enriched in digestion, IL-1 production, gastric acid secretion and so on. A total of 6 hub genes were generated by cytoHubba plug-in, among which ATP4A, CFTR and EPCAM had high diagnostic value. A total of 13 overlapping miRNA were predicted by 6 hub genes.

**Conclusion:**

ATP4A, CFTR and EPCAM may be potential biomarkers of CAG. hsa-miR-185-5p-CFTR, hsa-miR-4644-CFTR and hsa-miR-4505-CFTR are potential RNA regulatory pathways to control the progression of CAG disease. Finally, amonafide, etoposide, mycophenolate-mofetil, cycloheximide and Emetine may be potential therapeutic drugs for CAG.

## Introduction

Gastric cancer (GC) is one of the most common malignant tumors and the fourth leading cause of cancer-related death worldwide (1). Due to insidious disease progression in the early stage of GC, at the time of diagnosis most patient have advanced GC. Even if patients with advanced GC undergo surgery, the 5-year survival rate remains at a low level of 20-30% (2). Therefore, early diagnosis is the best way to improve the prognosis of GC (3). Studies have shown that the development of GC is a gradual process, and the Correa cascade reaction is considered to be the main pathway of GC occurrence (4).

Chronic atrophic gastritis(CAG) is considered to be the first step in a multi-step precancerous cascade, which is defined by replacement of appropriate gastric glandular structures with connective tissue (nonmetaplastic atrophy) or a different, non-native epithelium (metaplastic atrophy) on a background of chronic inflammation (5). A Meta-analysis showed that the annual incidence of CAG ranges from 0% to 11%, and a higher incidence can be observed in Helicobacter pylori (H.pylori)-positive individuals (6). It is estimated that, the annual risk of CAG progression to gastric adenocarcinoma is 0.1% to 0.3%, but may be higher in reality, depending on CAG severity, extent, concomitant intestinal metaplasia (IM), and other factors (5, 7). Early detection, early diagnosis and early treatment of CAG are effective means to prevent GC. At present, the “gold standard” of CAG diagnosis is still histological analysis of gastric biopsies obtained by upper gastrointestinal endoscopy, which is invasive, expensive and can not be used for extensive screening. It is important to note that there are currently no screening guidelines for general average risk population, which could be attributed to invasive nature of gold standard diagnostic modalities. In addition, because the distribution of CAG may be irregular, there is a risk of misdiagnosis caused by sampling errors. Therefore, it is crucial to study the potential molecular mechanism of CAG, to identify more effective biomarkers to detect the occurrence of CAG, and to explore effective methods to control and prevent CAG. Microarray analysis is increasingly used to explore disease epigenetics and screen effective biomarkers for disease diagnosis and treatment. The data set generated by microarray analysis is a powerful tool to explore the pathogenesis of the disease, and the integration of these databases is helpful for more in-depth study of the mechanism of the disease.

**Figure 1:**
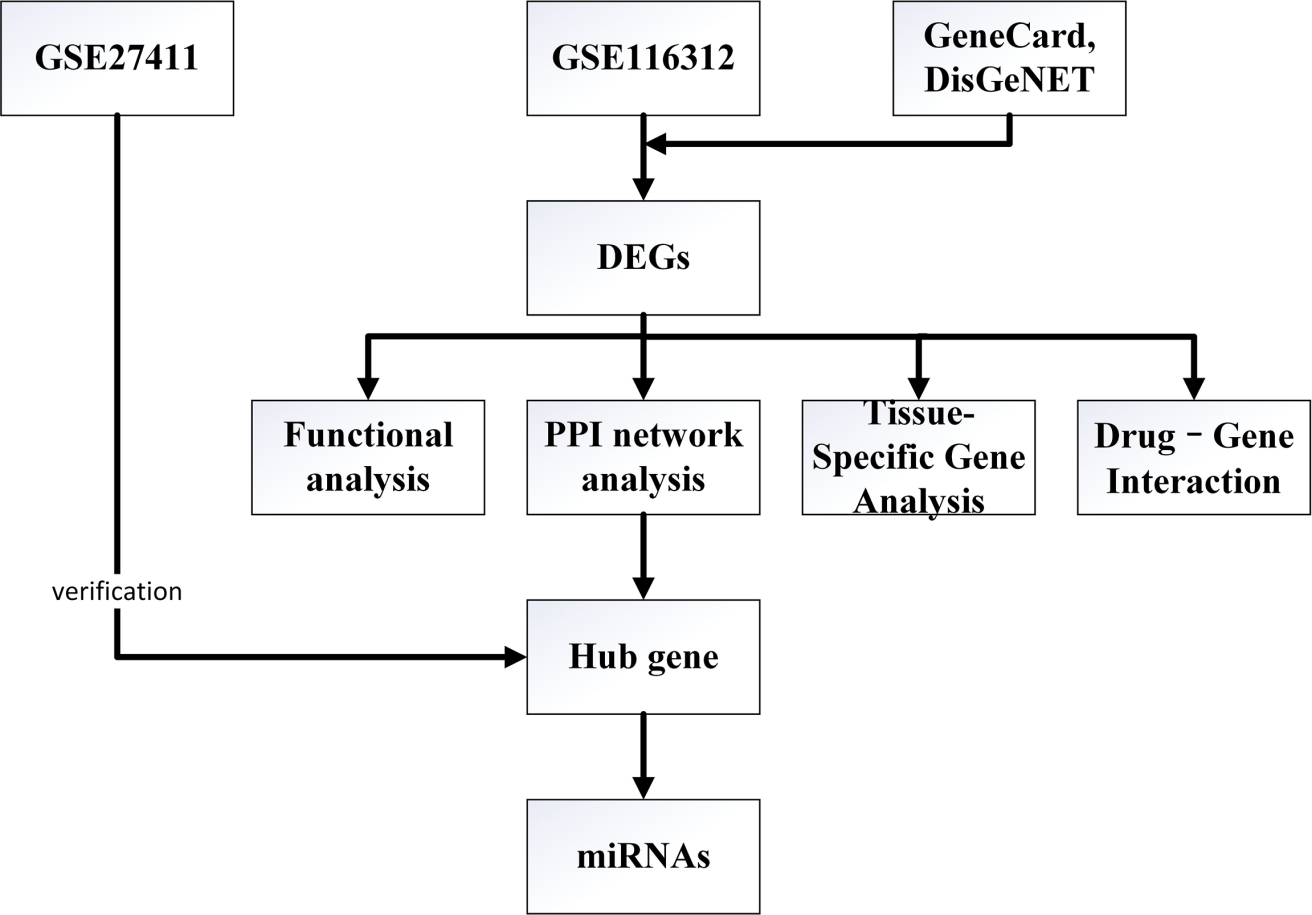
Flowchart of the study.

In this study, CAG-related microarray data sets were downloaded from GEO database, and DEGs were obtained by GEO2R online tool. We also searched for CAG-related genes through GeneCard and DisGeNET databases, and the final DEGs were obtained after the intersection of the CAG-related genes and the differentially expressed genes obtained from GEO database. The BioGPS database was used for query tissue specific gene expression. The DAVID database was used for enrichment analysis to understand the biological functions and pathways of DEGs. Protein-protein interaction (PPI) network was constructed using STRING database. Cytosacape software was used to analyze PPI network and screen hub gene. The CMap website was used to identify potential therapeutic CAG drugs. TargetScan, miRWalk and miRDB database were used to predict the upstream miRNA of hub gene. The specific process of this study is shown in **Figure 1**.

## Materials and methods

### Datasets collection

Two human microarray datasets of CAG were obtained by searching NCBI’s GEO database (http://www.ncbi.nlm.nih.gov/geo/). We downloaded the GSE116312 dataset for subsequent analysis and GSE27411 for validation. The GSE116312 dataset contains 9 samples (6 health samples and 3 CAG samples). The GSE27411 dataset contains 12 samples (6 health samples and 6 CAG samples).

### Screening of DEGs

GEO2R is an interactive network tool that can be used to identify differentially expressed genes under the same experimental conditions. We used GEO2R to analyze the differentially expressed genes between CAG and healthy people. The screening criteria was Adjusted P < 0.05and logFC > 1 or <-1. The GeneCard (https://www.genecards.org/) and DisGeNET (https://www.disgenet.org/) databases were used to predict CAG-related mRNA. After deleting the duplicate values, the Venn diagram was used to find out the overlapping mRNA of differentially expressed genes from the two databases and the GSE116312 dataset. And the overlapping mRNA were considered as final DEGs.

### Authentication of the tissue-specific expressed genes

BioGPS (http://biogps.org) is a centralized gene-annotation portal that enables researchers to access distributed gene annotation resources (8). In this study, BioGPS was used to analyze the specific expression of DEGs in organs and tissues.

Tissue-specific expressed genes should meet the following criteria:(1)the expression level of transcripts located in a single organ is more than 10 times the median;(2)the second highest expression organ does not exceed half of the highest expression level (9).

### Functional enrichment analysis

The Database for Annotation, Visualization and Integrated Discovery (DAVID, https://david.ncifcrf.gov/) (10) provides a comprehensive set of functional annotation tools for investigators to understand the biological meaning behind large lists of genes. In order to identify the biological function of DEGs, we used DAVID (version 6.8) to analyze DEGs by gene ontology (GO) and Kyoto Encyclopedia of Gene and Genome (KEGG) respectively. GO enrichment analysis includes biological processes (BP), cellular components (CC) and molecular functions (MF). P < 0.05 was considered to be statistically significant.

### Construction of PPI network and selection of hub gene

STRING (https://string-db.org) is a database of known and predicted protein-protein interactions (11). We used STRING database to build the PPI network of DEGs. The interaction score > 0.4 and removal of disconnected nodes were set to identify the crucial PPIs. Cytoscape software (version 3.9.1) was applied to display the relationship between proteins (12). The cytoHubba (13) plug-in in Cyto scape software is used to identify hub genes in the network. Maximal Clique Centrality (MCC), Maximum Neighborhood Component (MNC), Degree and Stress were used to calculate the first 10 central genes respectively, and the final hub gene was shown by Venn Diagram.

### Prediction of upstream miRNAs

Three databases, TargetScan (v7.0; https://www.targetscan.org) (14),miRDB(http://mirdb.org) (15), miRWalk (http://mirwalk.umm.uni-heidelberg.de) (16) were used to predict upstream miRNAs of hub genes. We choose miRNA which exists in the three at the same time as the target miRNA and build the miRNA-mRNA network.

### CMap analysis

Connectivity Map (17) (CMap, https://www.broadinstitute.org/connectivity-map-cmap) is to use small molecular drugs to treat the differences of gene expression in human cells, to establish a biological application database of small molecular drugs, gene expression and disease. After submitting the list of up-regulated and down-regulated genes as up-and down-regulated tags respectively, we estimated the connectivity score based on pattern matching algorithm to discover the functional links between drugs, genes and diseases through the instantaneous characteristics of common gene expression changes. The connectivity score from-1 to 1 is used to reflect the proximity between the expression profiles: a positive score indicates a promoting effect, while a negative score indicates an inhibitory effect.

## Result

### Identification of DEGs

**Figure 2:**
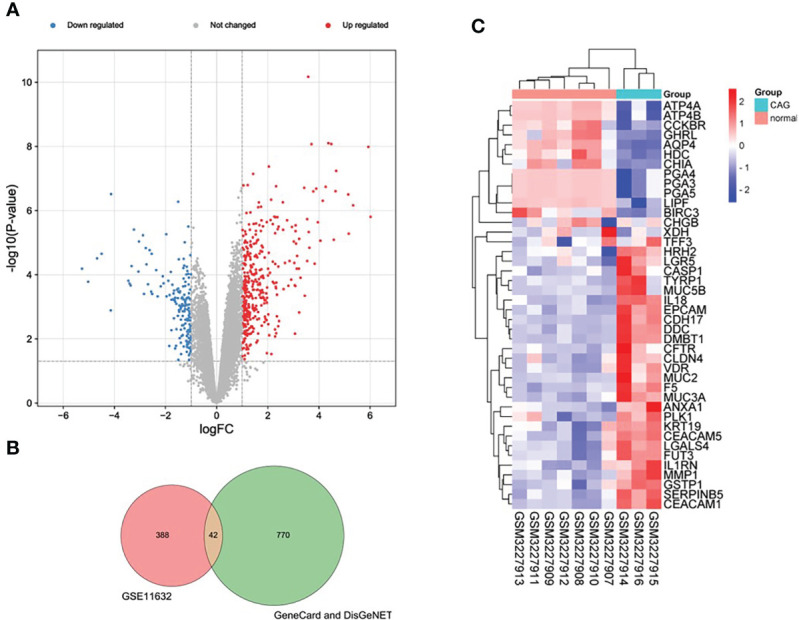
Expression profiles of DEGs in CAG. **(A)** Volcano plot of DEGs of GSE11632 (red: upregulated DEGs. green: downregulated DEGs.) **(B)** Venn diagram of DEGs from the two databases and the GSE116312 dataset. **(C)** Heatmap of final DEGs (red: high expression; blue: low expression).

According to the pre-set parameters, we screened 430 DEGs from the GSE11632 data set, including 315 up-regulated genes and 115 down-regulated genes. In addition, we obtained a total of 812 mRNA from GeneCard and DisGeNET databases. The intersection of the two sets of data produced 42 DEmRNA, (**Figure 2B**) including 27 up-regulated genes and 15 down-regulated genes. We plotted a heat map and a volcano map for data analysis and visualization (**Figures 2A**, **C**).

### Authentication of the tissue-specific expressed genes

A total of 24 tissue-specific genes were identified by BioGPS(**Table 1**). tissue-specific genes were mostly in the stomach (42%), followed by gut (21%), oral cavity, esophagus (13%), reproductive organs(8%), trachea (4%), pituitary (4%), placenta (4%) and liver (4%).

**Table 1:** Tissue-specific expressed genes identified by BioGPS.

Organ/tissue	Genes
Stomach	ATP4A、PGA4、GHRL、AQP4、CCKBR、LIPF、PGA3、ATP4B、CHIA、PGA5
Gut	CDH17、MUC2、XDH、LGALS4、CEACAM1
Oral cavity/ esophagus	VDR、IL1RN、CFTR
Reproductive organs	TYRP1、HDC
Trachea	LGR5
Pituitary	CHGB
Placenta	DMBT1
Liver	F5

### GO Term and KEGG pathway enrichment analysis of DEGs

**Figure 3:**
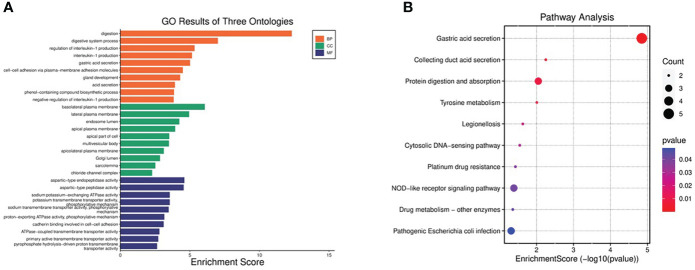
GO and KEGG pathway enrichment analyses of DEGs. **(A)** Top 10 enriched GO terms of the DEGs. **(B)** The bubble plot showing top 10 enriched KEGG pathways of DEGs.

Functional and pathway enrichment analysis of DEGs was performed by DAVID6.8 online tool. GO analysis showed that the biological process (BP) of DEGs mainly focused on digestion, digestive process, regulation of IL-1 production, IL-1 production, gastric acid secretion, cell-cell adhesion through plasma membrane adhesion molecules, gland development, acid secretion, biosynthesis of phenolic compounds, and negative regulation of IL-1 production (**Figure 3A**). The main cellular components (CC) include basolateral plasma membrane, lateral plasma membrane, inner body cavity, apical plasma membrane, apical part, polyvesicle, apical lateral plasma membrane, Golgi body cavity, sarcolemma, chloride channel complex (**Figure 3A**). Molecular function (MF) includes aspartate endopeptidase activity, aspartate peptidase activity, sodium: potassium exchange ATP activity, potassium transporter activity, phosphorylation mechanism, sodium transmembrane transporter activity, phosphorylation mechanism, proton output ATP enzyme activity, phosphorylation mechanism, cadherin binding, ATP enzyme coupled transmembrane transporter activity, primary active transmembrane transporter activity, Pyrophosphate hydrolysis drives the activity of proton transmembrane transporter(**Figure 3A**). KEGG pathway is mainly concentrated in gastric acid secretion, collecting duct acid secretion, protein digestion and absorption, tyrosine metabolism, Legionella disease, cytoplasmic DNA sensing pathway, platinum resistance, nod-like receptor signal pathway, drug metabolism-other enzymes, and pathogenic E. coli infection (**Figure 3B**).

### Construction of PPI network and selection of hub gene

**Figure 4:**
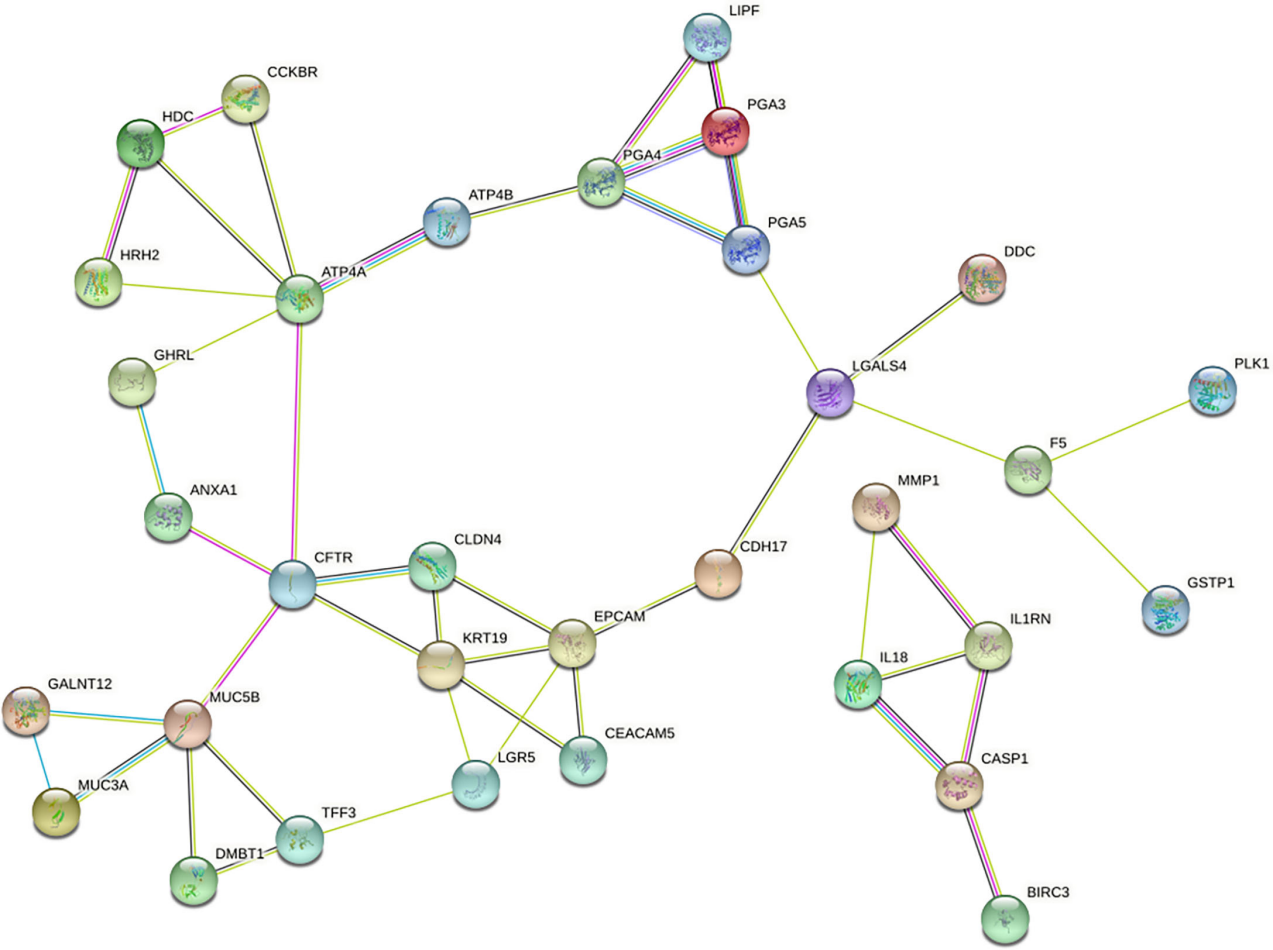
PPI network of DEGs constructed with STRING databases.

To further reveal the interaction between DEGs, we used STRING database to build a PPI network, which consists of 42 nodes and 47 sides (**Figure 4**). Then, the PPI network was analyzed by Cytoscape, and the four algorithms (MCC, MNC, Degreeand, Stress) of cytoHubba plug-in were used to identify hub genes. The intersection of the first 10 genes of the four algorithms was considered to be hub gene: ATP4A, CFTR, KRT19, PGA4, EPCAM, MUC5B.

### External verification and effectiveness evaluation

**Figure 5:**
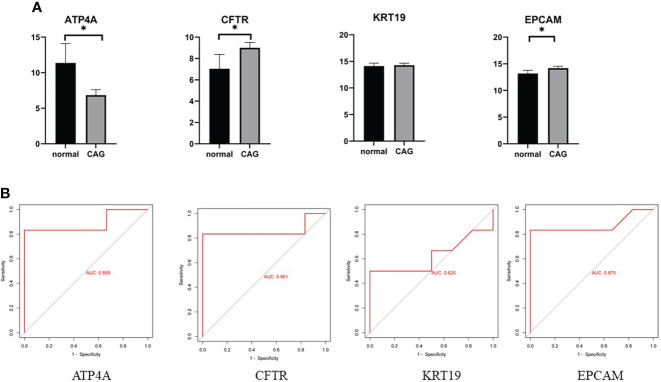
**(A)** Data validation of hub genes by GSE27411. *P < 0.05. **(B)** ROC curve of the 4 hub genes. AUC area under the ROC curve.

To further verify the reliability of our results, we used GSE27411 dataset to verify the expression of hub gene in normal samples and CAG samples (**Figure 5A**), and evaluated the diagnostic value of hub gene by receiver operating characteristic (ROC) curve (**Figure 5B**). The results showed that the differential expression of ATP4A, CFTR and EPCAM in the GSE27411 dataset was statistically significant, while the differential expression of KRT19 in the dataset was not statistically significant. (PGA4, MUC5B were not found in the dataset). ROC curve analysis showed that ATP4A, CFTR and EPCAM had good accuracy in the diagnosis of CAG. Therefore, these three hub genes can be used as potential biomarkers for CAG diagnosis.

### Construction of miRNA-mRNA network

**Figure 6:**
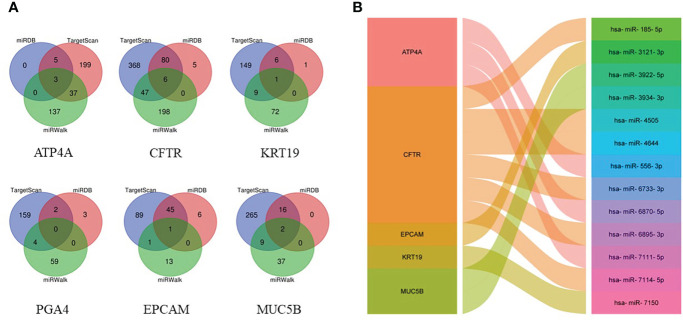
Prediction of targeted miRNAs. **(A)** Venn diagram of the common predicted miRNAs. **(B)** The miRNA-mRNA network constructed based on the predicted relationship.

By searching TargetScan, miRDB and miRWalk databases, we predicted a total of 13 overlapping miRNA related to the above hub genes (**Figure 6A**). MiRNA-mRNA network was established according to the corresponding relationship (**Figure 6B**).

### CMap analysis

After uploading DEGs to the CMap database, we obtained a list of compounds. And we listed the top five compounds with the highest scores for potential therapeutic drugs for CAG in **Table 2**.

**Table 2:** The top five compounds with the highest scores in the CMap analysis.

Name	ID	Description	Score
amonafide	BRD-K56334280	Topoisomerase inhibitor	-97.5
etoposide	BRD-K37798499	Topoisomerase inhibitor	-95.67
mycophenolate-mofetil	BRD-K92428153	Dehydrogenase inhibitor	-95.25
cycloheximide	BRD-A62184259	Protein synthesis inhibitor	-95.03
emetine	BRD-A25687296	Protein synthesis inhibitor	-94.79

## Discuss

Although important progress has been made in the treatment of GC, the overall survival rate of GC is still very low (1). CAG is one of the precancerous stages of intestinal-type gastric cancer. Studies have shown that 90% of gastric epithelial malignant tumors are caused by CAG (18). The precancerous cascade development of GC is widely recognized: from atrophic gastritis (AG) to gastrointestinal metaplasia (GIM), low-grade atypical hyperplasia (LGD), high-grade atypical hyperplasia (HGD), and eventual cancer (3). In Japan, more than 50% of GC are diagnosed at an early stage through organized screening. Early-stage GC patients have a good prognosis, with a >90% 5-year survival rate (19). Therefore, early detection and blocking the development of CAG are effective measures for the prevention and treatment of GC. Common screening methods for CAG include gastroscopy, imaging examination and biomarker examination. Due to the lack of specificity of clinical manifestations in CAG, the efficiency of imaging examination is not obvious. The diagnosis and follow-up of CAG depend on endoscopy and histopathological assessment. Endoscopy also has some limitations due to the risk of invasive operation, time-consuming (20), high price and stringent requirements of endoscopic physicians. In addition, although gastrin-17(G-17) and Pepsinogen (PG) I or II may be helpful for determining atrophy existence and location. But it had not been widely used with low stability cases (21). Therefore, it is urgent to investigate some non-invasive, sensitive and reliable biomarkers to enrich the diagnostic methods of CAG. This study attempts to discover CAG biomarkers and potential therapeutic drugs through bioinformatics analysis. Our results are expected to provide new insights into the pathogenesis and therapeutic targets of CAG.

In this study, we identified 42 DEGs, including 24 tissue-specific genes. GO enrichment analysis showed that up-regulated DEGs were mainly involved in inflammatory responses and pro-inflammatory effects (such as regulation of IL-1 production, IL-1 production). Interleukin-1 (IL-1) is a pro-inflammatory cytokine with a variety of biological effects (22). As a potent pro-inflammatory cytokine, it participates in a variety of cellular activities, including cell proliferation, differentiation and apoptosis. Among physiological functions related to stomach, IL-1 β is a potent inhibitor of gastric acid secretion (23). Studies have shown that the high expression of IL-1 β may inhibit the production of gastric acid and weaken the inhibition of H.pylori infection, which leads to CAG (24). Down-regulated DEGs is mainly involved in digestion, gastric acid secretion and other processes. KEGG pathway is mainly enriched in gastric acid secretion, protein digestion and absorption, and nod-like receptor signal pathway. To further explore the pathogenesis of CAG, we obtained six hub genes through four algorithms of cytoHubba plug-in in Cytoscape software: ATP4A, CFTR, KRT19, PGA4, EPCAM, MUC5B.

The protein encoded by ATP4A belongs to a family of P-type cation-transporting ATPases. The gastric H^+^, K^+^-ATPase is a heterodimer consisting.This enzyme is a proton pump that catalyzes the hydrolysis of ATP coupled with the exchange of H(+) and K(+) ions across the plasma membrane. In terms of expression, ATP4A restricts expression to the stomach (25). Some studies have shown that the mutation of ATP4A is related to the occurrence of GC (26, 27).

CFTR is a transmembrane transporter that acts as a chloride channel, regulates the activity of epithelial sodium channels and other channels, and plays an important role in controlling the secretion and absorption of ions and water in epithelial tissue and humoral homeostasis (28). The protein encoded by KRT19 is a member of the keratin family. Keratin is a protein in the intermediate filament of epithelial cells, which has been used as a specific marker of epithelial-derived tumor cells (29). Previous studies have shown that KRT19 can not only be used to detect lymph node metastasis of GC (30), but also has potential diagnostic value for the prognosis of GC (31). PGA4 is the precursor of pepsin. It has been suggested that pepsinogen levels in serum may serve as an atrophic gastritis marker, but there is currently no high-quality evidence to demonstrate its high diagnostic value in CAG (32). EPCAM is a transmembrane glycoprotein expressed in a variety of epithelial tissues. EPCAM is one of the tumor stem cell markers (33). A meta-analysis showed that overexpressed EPCAM in GC was associated with tumor size, lymph node metastasis and poor prognosis (34). MUC5B is a member of the mucin family. Mucins in gastric mucus are glycoproteins that protect gastric mucosa. Differences in mucin expression in precancerous lesions have been reported (35). MUC5B is not expressed in normal gastric tissue, but overexpressed in GC and some subtypes of colorectal cancer. In the MUC5B knockdown model, the proliferation, migration and invasion of human gastrointestinal cancer cells were significantly affected (36).

We completed the validation of 4 of these genes (ATP4A, CFTR, KRT19, EPCAM) in the GSE27411 dataset, and we observed the differential expression of ATP4A, CFTR, and EPCAM with statistical significance (P<0.05). The ROC curve analysis shows that these genes have high diagnostic value for CAG. Interestingly, ATP4A is specifically expressed in gastric organs, so ATP4A may be the most potential biomarker for CAG.

MiRNA is a small, non-coding, single-stranded RNA molecule consisting of about 19-23 nucleotides (37). MiRNA regulates gene expression mainly by degrading mRNA or inhibiting its translation (38). Studies have shown that miRNA is widely involved in the generation and development of tumors (39). A total of 13 upstream overlapping miRNAs of hub gene were predicted by searching TargetScan, miRDB and miRWalk database. Except hsa-miR-185-5p, hsa-miR-4644 and hsa-miR-4505, the remaining 10 miRNAs have no related research reports. Interestingly, hsa-miR185-5p, hsa-miR-4644 and hsa-miR-4505 all target CFTR. Therefore, we believe that hsa-miR185-5p-CFTR, hsa-miR-4644-CFTR and hsa-miR-4505-CFTR regulatory networks play an important role in the pathogenesis of CAG. And hsa-mir-185-5p has been observed to be down-regulated in GC tissue (40) and validated as an independent prognostic factor (41). High expression of hsa-miR-4644 has been observed in other tumors (42), but there have been no studies on the stomach. There are few researches on hsa-miR-4505.

Based on CMap database analysis, we obtained 5 potential CAG therapeutic compounds. Amonafide and etoposide are topoisomerase II inhibitors that are commonly used chemotherapeutic drugs. Amonafide and etoposide induce apoptotic signaling by blocking the binding of Topo II to DNA (43, 44), which suggests that their therapeutic value in CAG is worth exploring. Mycophenolate-mofetil is a selective immunosuppressant, which is commonly used to prevent organ transplant rejection and autoimmune diseases such as systemic lupus erythematosus. Mycophenolic acid, the metabolite and active component of mycophenolate-mofetil, can inhibit the migration and invasion of GC cells through various molecular pathways (45, 46). Cycloheximid is an eukaryotic protein synthesis inhibitor, and is the most commonly used laboratory reagent to inhibit protein synthesis (47). Emetine is also a protein synthesis inhibitor, with anti-malaria, anti-amoeba effects (48). In addition, emetine also exhibits anti-leukemia effect (49), its therapeutic value in CAG remains to be further explored.

In this study, we identified six hub genes of CAG. Among them, ATP4A, CFTR and EPCAM have high diagnostic value and can be used as potential biomarkers of CAG. Our results are expected to provide valuable guidance for the diagnosis of CAG. However, our study also has certain limitations, because the results of this study are only based on bioinformatics analysis and require further experimental and clinical validation.

## Conclusion

In conclusion, we found that ATP4A, CFTR and EPCAM have high diagnostic value and can be used as potential biomarkers of CAG. Furthermore, we propose that hsa-miR-185-5p-CFTR, hsa-miR-4644-CFTR and hsa-miR-4505-CFTR are potential RNA regulatory pathways to control the progression of CAG disease. Finally, we provide 5 compounds that may treat CAG.

## Data availability statement

The original contributions presented in the study are included in the article/**Supplementary Material**. Further inquiries can be directed to the corresponding author.

## Ethics statement

Ethical review and approval was not required for the study on human participants in accordance with the local legislation and institutional requirements. Written informed consent for participation was not required for this study in accordance with the national legislation and the institutional requirements.

## Author contributions

BS and XL conceptualized the study design. BS and QC drafted the manuscript. SF revised the manuscript. BS, TL, YL, and QS collected data and performed the analysis. All authors contributed to the article and approved the submitted version.

## Funding

This study was supported by the Natural Science Foundation of Anhui Province (No. 2008085MH265) and the Chinese and Western Medicine Cooperation Project for Difficult and Complicated Diseases of Anhui Province: Synergistic Treatment of Chronic Atrophic Gastritis with Intestinal Metaplasia Based on Chinese and Western Medicine (No. 0708–2021).

## Conflict of interest

The authors declare that the research was conducted in the absence of any commercial or financial relationships that could be construed as a potential conflict of interest.

## Publisher’s note

All claims expressed in this article are solely those of the authors and do not necessarily represent those of their affiliated organizations, or those of the publisher, the editors and the reviewers. Any product that may be evaluated in this article, or claim that may be made by its manufacturer, is not guaranteed or endorsed by the publisher.
